# The effectiveness and safety of rectal modular dissection for male middle and low rectal cancer after neoadjuvant chemoradiation therapy: the short-term outcome

**DOI:** 10.7150/jca.91776

**Published:** 2024-01-01

**Authors:** Weijie Chen, Yuxin Liu, Yang An, Xiaoyuan Qiu, Jiaolin Zhou, Lin Cong, Guole Lin

**Affiliations:** Department of General Surgery, Peking Union Medical College Hospital, Chinese Academy of Medical Sciences, Shuaifuyuan 1#, Beijing 100730, P. R. China.

**Keywords:** rectal cancer, total mesorectal excision, rectal mobilization, clinical trial

## Abstract

**Background:** The purpose of this study was to assess the efficacy and safety of rectal modular dissection (RMD) in male patients with middle and low rectal cancer. RMD is a technique used to guide the surgical procedure for rectal mobilization, with the ultimate goal of achieving total mesorectal excision. In order to evaluate the effectiveness of RMD, a single-center, non-inferiority randomized clinical trial was carried out.

**Methods:** Eligible patients were randomly assigned into two groups: the RMD group and the traditional rectal mobilization (TRM) group. Demographic characteristics, perioperative data and pathological results of the surgical specimens were collected for analysis. additionally, assessments of urogenital function and defecation function were conducted for all participants.

**Results:** A total of 103 patients (RMD group 53 patients and TRM group 50 patients) were included to analyzed. There were no significant differences in age, body mass index, ASA classification, and tumor characteristics between two groups. The RMD group had significantly lower blood loss (*P* = 0.00), shorter operative duration (*P* = 0.00), and shorter hospital stay (*P* = 0.04) compared to the TRM group. The complete rate of mesorectal excision was higher in the RMD group (98.1%) compared to the TRM group (86.0%, *P* = 0.02). In terms of functional outcomes, the RMD group had better evaluation scores for urethral function (IPSS score, *P* = 0.01), erectile function (IIEF-5 score, *P* = 0.00) and defecation function (LARS score, *P* = 0.00) at the one-year postoperative follow-up. The 1-year disease-free survival rate was similar between the two groups (*P* = 0.28).

**Conclusions:** These results suggest that RMD is an effective and safe approach for achieving total mesorectal excision while promoting better functional outcomes for patients.

The trial was registered in Chinese Clinical Trial Registry (ChiCTR2100052094).

## Introduction

Rectal cancer poses a significant burden in terms of incidence and mortality rates. In the United States alone, over 43,000 new cases of rectal cancer are diagnosed annually, with approximately 53,200 combined deaths from rectal and colon cancer 1. Currently, neoadjuvant chemoradiotherapy followed by radical resection is the recommended treatment approach for advanced rectal cancer, aimed at improving patient outcomes 2. However, the use of preoperative chemoradiotherapy often leads to tissue edema, which can complicate surgical procedures and increase the risk of complications. Additionally, in male patients, the low rectum is in close proximity to the prostate, seminal vesicle, and has poor blood supply. Despite advancements in technology, the treatment of low rectal cancer and the prevention of associated complications remain ongoing challenges for clinicians. Furthermore, the proximity of the low rectum to critical structures can impact urinary and erectile function, and the risk of leakage increases with more distal anastomoses 3. These factors highlight the importance of developing effective strategies to address the treatment of low rectal cancer and mitigate potential complications.

Total mesorectal excision (TME) is a widely accepted surgical principle for the treatment of rectal cancer. It was first described by Heald in 1982 and has since become the gold standard approach. TME involves the en-bloc removal of the rectal cancer, rectum, and perirectal tissues, ensuring complete resection of the tumor along with the surrounding mesorectal envelope. This technique aims to achieve negative lateral margins and circumferential margins histologically. Despite its effectiveness, there is currently no standardized procedure or operational guide for performing TME. The lack of a standardized approach can lead to variations in surgical techniques and outcomes. Therefore, there is a need for further research and development of guidelines to provide surgeons with a clear and consistent framework for performing TME. This would help ensure the optimal implementation of this surgical principle and improve outcomes for patients with rectal cancer.

In our study, we have modified the procedure of laparoscopic rectal mobilization based on the radical advanced modular pancreatectomy and splenectomy (RAMPS) technique introduced in 2003 4. We have named this modified approach rectal modular dissection (RMD), which allows for the achievement of standard total mesorectal excision (TME). The RMD approach begins by accessing the retrorectal space posteriorly in the midline. This area, known as the "Holy plane," has minimal vessel and nerve distribution between the rectal fascia propria and the parietal pelvic fascia. We then extend the space laterally by cutting the lateral ligaments, which contain branches of the middle rectal artery and venous plexus. By creating the posterior and lateral spaces, we can easily expose the anterior Denonvilliers' fascia and protect the prostate, seminal vesicles, and Neurovascular Bundle (NVB). The objective of our study is to evaluate the effectiveness and safety of the RMD technique in male patients with middle and low rectal cancer. In this report, we present the short-term outcomes of our trial.

## Method

### Study design and participants

The study was designed as a single-center, randomized, prospective, and single-blind noninferiority trial. It was registered in the Chinese Clinical Trial Registry under the registration number ChiCTR2100052094. The eligible participants for the study were male patients with middle (≤ 10 cm from the anal verge) or low (≤ 5 cm from the anal verge) rectal adenocarcinoma, without evidence of distant metastases, who required laparoscopic anterior resection of the rectum. Additional inclusion criteria included being ≤ 70 years old, having an American Society of Anesthesiologists (ASA) classification of 1 to 3, histologically confirmed primary adenocarcinoma, T3, T4, or node-positive tumors confirmed by MRI or endorectal ultrasound with accepted preoperative chemoradiotherapy, and willingness to sign informed consent. The preoperative chemotherapy were XELOX or capecitabine alone. The regimen of XELOX was termed as the combination of capecitabine (1000 mg/m2 twice daily for 14 days of every 21-day cycle) plus oxaliplatin (130 mg/m2 intravenously on day 1). Concurrently, radiotherapy (50 Gy total administered over 25 fractions) last for approximately five weeks. Patients were restaged 6 to 8 weeks after chemoradiotherapy, and those with an incomplete response had TME.

Exclusion criteria consisted of having undergone total or partial pelvic exenteration, diagnosed urinary system disease (including urinary tract infections and neurological bladder dysfunction), prostate disease (such as benign prostatic hyperplasia), serious sexual dysfunction, and the use of drugs that could affect bladder, sexual, or defecation functions. Patients who underwent local resection, R2 resection, additional pelvic surgery (such as ureterectomy, prostatectomy, or ureteral double-J stenting), or had their surgery transferred to Miles' surgery or required emergency surgery were also excluded from the trial. All patients provided informed consent. Demographic information, co-morbidities, and medical history of the participants were collected. The study was approved by the Ethics Committee of Peking Union Medical College Hospital (Ethic review approval No. JS-3361).

### Randomization and blinding

Patients were randomly assigned to either the RMD group or the traditional rectal mobilization (TRM) group in a 1:1 ratio. The randomization process was conducted by drawing lots. When a researcher logged into the research system and uploaded the patient's information, the research team members would draw lots to determine the group assignment. To ensure unbiased assessment, both the patients and the investigators involved in perioperative assessment and follow-up were masked or blinded to the randomization outcomes. This means that they were unaware of which group the patients were assigned to. However, the surgeons who performed the operations were not masked to the treatment assignment, as they needed to know the specific procedure to be performed.

### Procedures

**Figure 1:**
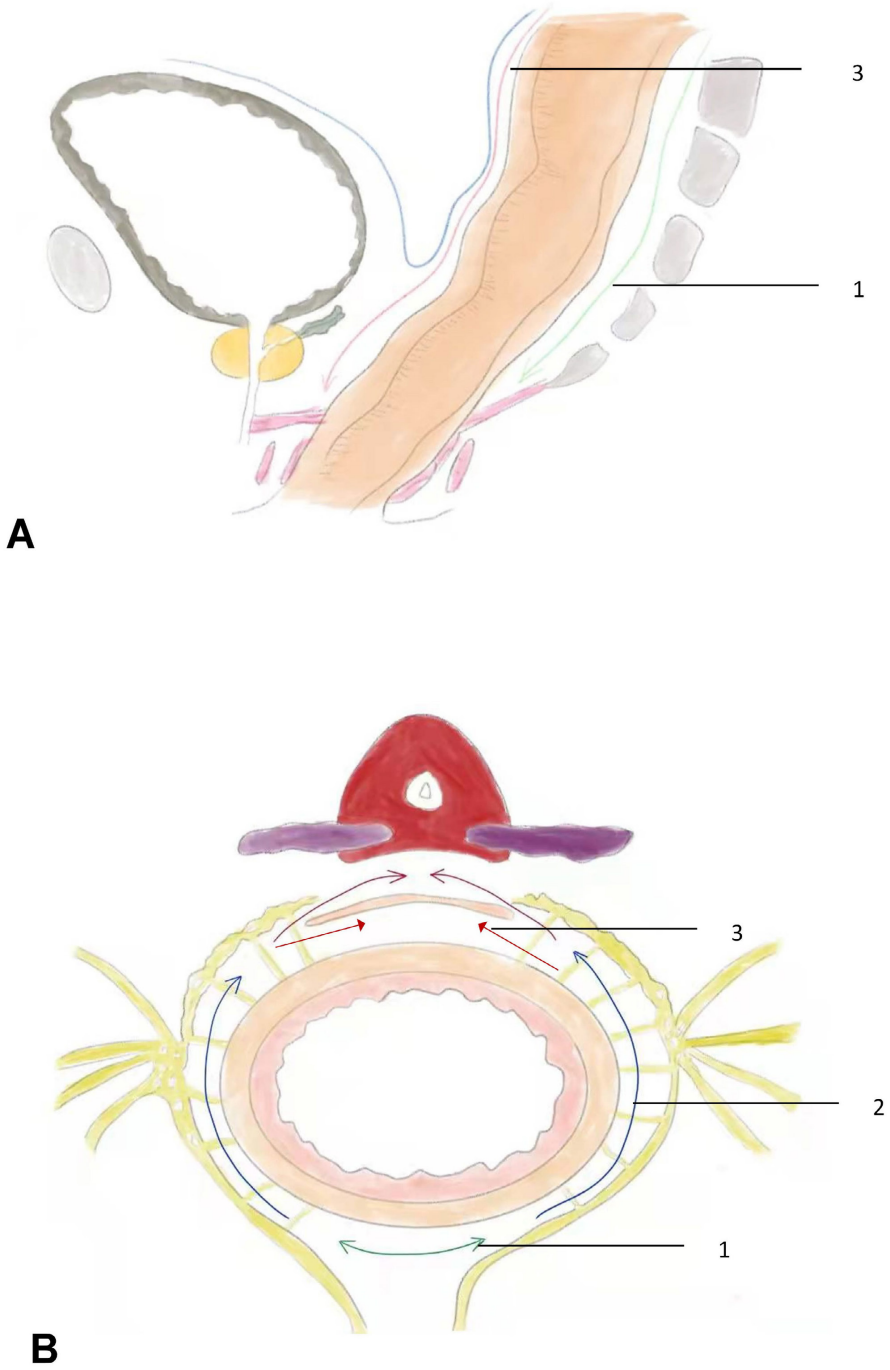
the sketch of rectal modular dissection. **A,** the sagittal view of operative field. **B,** the cross-section view of operative field. Step 1, follow the green line into the retrorectal space. Step 2, follow the path indicated by the blue line to incise the lateral ligaments. Step3, the red line represents the path is to incise the anterior plane, which indicates the direction of the incision in the front part of the rectum. Denonvilliers' fascia, which is a double-layered fascia between the rectum and seminal vesicles, can be removed along the purple line. However, it can also be preserved by cutting along the red line, indicating a more careful and precise dissection to spare the fascia.

The surgical procedures for RMD were performed as follows: (1) Skeletonization of the inferior mesenteric artery and severing the superior rectal blood vessel. The sigmoid colon was gently retracted anteriorly to identify the avascular "Holy fascia." The retrorectal space was accessed in the midline, and an ultrasonic scalpel or electrocautery was used to expand the retrorectal space distally to approximately 3 cm from the tumor margin. (2) Expansion of the retrorectal space laterally to the lateral ligaments: The lateral ligaments, which contain the middle hemorrhoidal vascular pedicles, were incised. The retrorectal space was further expanded laterally, and dissection was continued distally to approximately 3 cm from the tumor margin or the pelvic diaphragm. (3) Incision of the peritoneum anterior to the rectum: The peritoneum was incised approximately 0.5 cm above the fold of the cul-de-sac. The dissection plane was continued close to the anterior surface of the rectum while keeping Denonvilliers' fascia intact. This allowed for the preservation of the seminal vesicles, prostate capsule, and Neurovascular Bundle (NVB). In cases where the rectal cancer was located anteriorly, Denonvilliers' fascia was separated from the prostate capsule using sharp dissection with an ultrasonic scalpel to ensure a clear margin (Figure 1).

The main difference between the RMD and TRM procedure was that the procedure sequence strictly followed from a back-to-bilateral-to-front approach under laparoscopy (Supplementary videos 1 and 2). The colorectal or coloanal anastomosis method was standardized using a circular stapler. Additionally, all patients had right and left pelvic drainage tubes placed near the anastomotic site. Surgeons were required to have experience performing at least 100 laparoscopic anterior resections of the rectum, as well as more than 20 RMD and 20 TRM procedures for rectal cancer. The unedited videos of 2 RMD procedures and 2 TRM procedures performed by the surgeons were reviewed by the quality control committee to assess their surgical skills in accordance with the protocol requirements. Surgical videos of all patients were stored for random inspection by the quality control committee.

For low rectal cancer cases, a preventive stoma was created. Additionally, all patients had two drainage tubes placed near the pelvic anastomosis, which were removed on the third day after surgery. The amount of individual blood loss and the duration of the operation were recorded. Perioperative complications such as anastomotic leakage, postoperative hemorrhage, and incision infection were also documented.

### Pathological assessment

The surgical videos and photographs of the resected specimens were preserved for quality assessment by the quality control committee. In accordance with the American Joint Committee on Cancer 7th edition TNM staging system, dedicated pathologists conducted a comprehensive evaluation of the TNM category of the specimens. Additionally, the total number of resected lymph nodes was counted, and the integrity of the mesorectum was graded 5. The positive rate of circumferential resection margin (CRM) was compared between the groups.

### Urogenital functions

The urethral catheter was removed on the 6th day after surgery for patients with low rectal cancer, and on the 2nd day for patients with middle rectal cancer. If patients experienced difficulty urinating, a bladder ultrasound was performed. If the postvoid residual urine exceeded 100 ml within 1 day of catheter removal, urinary retention was diagnosed and a urethral tube was reinserted 6. To compare urinary function between the groups, the International Prostate Symptom Score (IPSS) was utilized. The IPSS includes items related to emptying, frequency, intermittency, urgency, weak stream, hesitancy, and nocturia 7. Each item is assigned a score ranging from 1 to 5, with higher scores indicating more severe urinary dysfunction.

The male erectile function of patients in both groups was also compared using the International Index of Erectile Function (IIEF-5) scale. This scale consists of 5 items, including confidence in erectile function, maintaining an erection, success rate of insertion, success rate of sexual intercourse, and satisfaction rating 8. Each item is scored from 0 to 5 points, with higher scores indicating better sexual function.

### Defecation functions

The bowel dysfunction following a low anterior resection was assessed using the low anterior resection syndrome (LARS) score. This score consists of 5 items: incontinence for flatus and liquid stools, defecation frequency, stool clustering, and urgency 9. The score ranges from 0 to 42, with a lower score indicating better defecation function. The scores are categorized as follows: no LARS (0-20), minor LARS (21-29), and major LARS (30-42).

### Follow-up

These patients received adjuvant therapy and underwent standard follow-up at the outpatient department. Clinical examinations and serum tumor marker evaluations were conducted every 3 months, while CT examinations of the chest, abdomen, and pelvis were performed every 6 months during the first 2 years. Tumor relapse was defined as the occurrence of distant metastases or local relapse, including central (presacral, anastomotic, and perineal sites) and lateral pelvic sites. If patients did not experience tumor relapse, the study endpoint was set at 3 years.

### Statistical analysis

Continuous variables were presented as mean ± standard deviation and analyzed using the t-test to compare differences between groups. Categorical variables were presented as numbers (percentages) and analyzed using the chi-squared test or Fisher's exact test. A two-sided *P*-value of less than 0.05 was considered statistically significant. Statistical analyses were performed using SPSS for Windows, version 20.0 (SPSS, Chicago, IL, USA). We used the formula n = 2 * [(Zα/2 + Zβ) * σ / Δ]² to calculate the sample size (independent samples t-test for equal sample sizes). According to our preliminary observations, the σ of IIEF-5 score was 3 and the Δ was1.2. The expected sample size should be more than 42.

## Results

### Demographic characteristics of included patients

**Figure 2:**
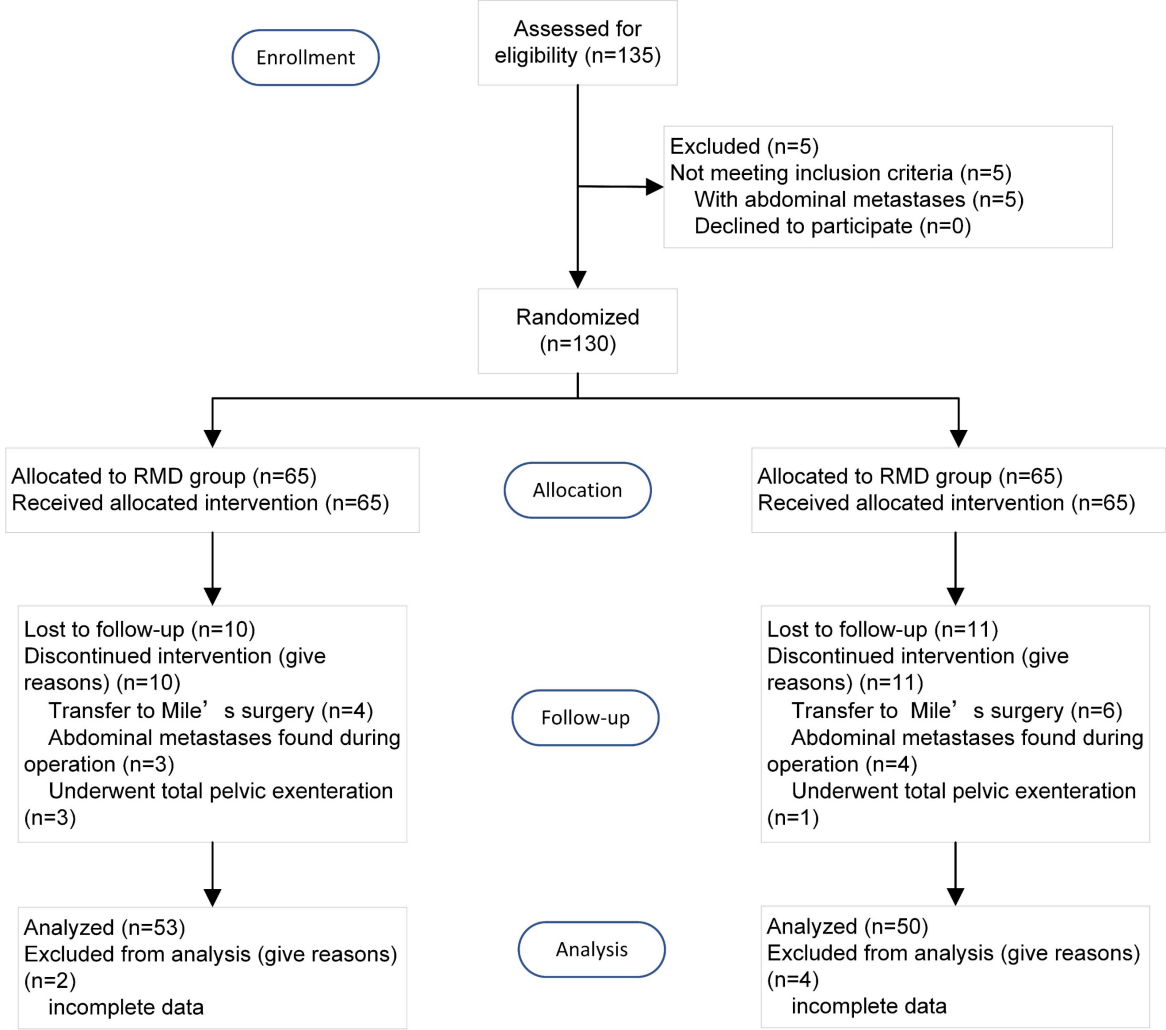
The flow chart of the study

Between January 1, 2020, and January 1, 2022, a total of 115 patients were enrolled in the study. And 3 patients were excluded for abdominal metastases. 112 patients were included in the study and randomly assigned to either the RMD group (56 patients) or the TRM group (56 patients). During the peri-operation and follow-up period, 3 patients were transferred to Mile's surgery, 3 patients were found metastases during operation, 2 patients underwent total pelvic exenteration, and 1 patient lost to follow-up. Ultimately, data of 103 patients were analyzed (RMD group 53 patients and TRM group 50 patients). The flow chart depicting the patient selection process is presented in Figure 2. The demographic characteristics of the included patients are summarized in Table 1.

The average age of included patients were 58.7 years. There were no significant differences between the two groups in terms of age (*P* = 0.90), body mass index (*P* = 0.19) and ASA classification (*P* = 0.19). Additionally, there were no statistically significant differences observed in tumor size (*P* = 0.53), chemotherapy regimen (*P* = 0.75), ypT category (*P* = 0.33), ypN category (*P* = 0.83), tumor site (*P* = 0.56) and distance from the anal verge (*P* = 0.62) between the two groups.

### Perioperative outcomes

During the operation, the RMD patients had significantly less blood loss compared to the TRM patients (35.9 ± 20.2 ml vs. 83.6 ± 27.5 ml, *P* = 0.00, as shown in Table 2). Additionally, the operative duration in the RMD group was significantly shorter than that in the TRM group (127.9 ± 27.4 mins vs. 143.8 ± 24.6 mins, *P* = 0.00). In patients with low rectal cancer, a preventive stoma was performed. Subsequently, 20 patients (37.7%) in the RMD group and 16 patients (32.0%) in the TRM group received a preventive stoma.

During the perioperative period, 2 patients (3.8%) in the RMD group and 4 patients (8.0%) in the TRM group experienced anastomotic leakage. Urine retention occurred in 2 patients (3.8%) in the RMD group and 5 patients (10.0%) in the TRM group. In the TRM group 1 patient (2.0%) experienced ileus and 1 patient (2.0%) had a stoma-related complication. No other complications, such as myocardial infarction, cerebral infarction, deep vein thrombosis, pulmonary embolism, pulmonary infection, and acute renal failure, were reported. According to the Clavien-Dindo classification, 4 patients (7.5%) in the RMD group and 8 patients (16.0%) in TRM group were classified as grade II, the complications included anastomotic leakage, stoma-related complications, urine retention and ileus. All of them were improved after conservative treatment. While the complications of 3 patients (6.0%) in the TRM group were classified as III-IV grade. These three patients suffered anastomotic leakage, 1 patient had to receive a delayed stoma and 2 patients were treated with a drainage tube under local anesthesia. The incidence of complications was significantly lower in the RMD group compare to the TRM group (*P* = 0.04). However, there was no significant differences in the composition of complications (*P* = 0.88) or their severity (*P* = 0.24) between the two groups.

The average length of hospital stay for the RMD group was 7.0 ± 1.3 days, while for the TRM group it was 7.6 ± 1.7 days. This indicates that the average length of hospitalization for the RMD group was shorter than that of the TRM group (*P* = 0.04).

### Pathological assessment

**Figure 3:**
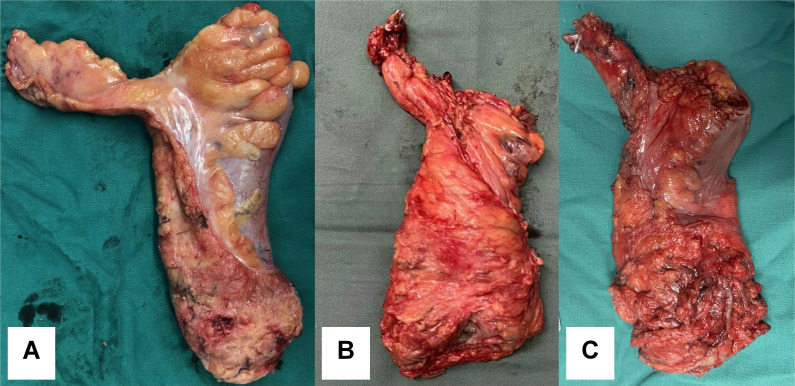
Specimen complete grading. A, complete. B, nearly complete. C, incomplete.

The margin clearance of resected specimen was similar between the RMD group and the TRM group. The rate of CRM positivity did not show a significant difference between the two groups (*P* = 0.08, as shown in Table 3). However, the mesorectum of the specimen in the RMD group was more complete compared to that in the TRM group (98.1% vs. 86.0%, *P* = 0.02). The integrity quality of resected specimen was classified into three subtypes: incomplete, nearly complete and complete (as shown in Figure 3). The specimen integrity grading of the RMD group was also higher than that of the TRM group (*P* = 0.02). Additionally, the number of periintestinal lymph nodes harvested by the RMD procedure was higher than that by the TRM procedure (14.1 ± 5.5 vs. 12.0 ± 4.2, *P* = 0.03).

The postoperative pathological results revealed that the proportion of tumor differentiation (well, moderate and poor) was similar between the two groups (*P* = 0.51, as shown in Table 4). Besides, there was no significant difference observed in the pathological T category (*P* = 0.78) and in pathological N category (*P* = 0.69) between the two group groups.

### Follow-up

All patients in both groups were followed up more than one year. Among them, 16 patients in the RMD group and 21 patients in the TRM group were followed up for more than two years. At the one-year postoperative follow-up, 1 patient in the RMD group and 3 patients in the TRM group were found to have local recurrence. However, no patient died or experienced distant metastases. The one-year disease-free survival rate between the two groups remained similar (98.1% vs 94.0%, *P* = 0.28), and all patients continued to be followed up.

Urethral function, erectile function and bowel function were assessed using IPSS, IIEF-5 and LARS questionnaire before surgery and one year after the surgery. Before surgery, there were no significant differences observed between the two groups in terms of IPSS score (1.4 ± 1.3 vs. 1.0 ± 1.2, *P* = 0.16), IIEF-5 score (19.9 ± 2.4 vs. 20.5 ± 1.6, *P* = 0.16) and LARS score (0.4 ± 1.6 vs. 0.7 ± 1.8, *P* = 0.51). However, at the one-year postoperative follow-up, the RMD group had a lower IPSS score compared to the TRM group (2.4 ± 1.7 vs. 3.5 ± 2.7, *P* = 0.01), a higher IIEF-5 score compared to the TRM group (19.8 ± 3.2 vs. 17.4 ± 1.7, *P* = 0.00), and a lower LARS score compare to the TRM group (14.5 ± 7.7 vs. 21.5 ± 7.0, *P* = 0.00).

## Discussion

In 1972, Heald et al. introduced the concept of total mesorectal excision (TME), which has significantly improved the treatment outcomes for rectal cancer over the past few decades. With the implementation of TME and the introduction of neoadjuvant chemoradiation therapy, the five-year local recurrence rate has decreased to 5%-10% 10. The RMD was developed to achieve a standardized TME procedure and minimize the challenges associated with the surgery. Particularly after neoadjuvant treatment, the inflammation response, tissue adhesion, edema, and obscured fascial planes resulting from chemoradiotherapy can make the operation more difficult. The use of the back-bilateral-front continuous navigation method or RMD technique can provide a safe and effective approach to guide the surgical procedure.

Before surgery, there were no significant differences between the RMD group and the TRM group in terms of the demographic characteristics, body mass index, ASA classification, preoperative treatment and tumor characteristics. However, during the surgery, the RMD group experienced significantly less blood loss and shorter operative durations compared to the TRM group. The RMD procedure detailed the surgical sequence and anatomical level clearly. The “Holy plane” between the anterior pelvic fascia and the fascia propria of rectum is accessed first, as it contains fewer blood vessel and nerve distributions. The fascia propria of rectum encases the rectum and its blood vessels, nerve supply and lymphatic tissues. The Mobilization of the rectum is performed outside of the fascia propria of rectum to avoid avulsion of the rectal vessels and posterior rectal wall. Surgeons must also be cautious not to damage the sacral plexus veins and middle sacral artery. The avascular presacral space is then extended to the lateral ligaments, which may contain middle rectal artery branches and venous plexus in about 25% of patients. Exposing the retrorectal space can facilitate the cutting of the lateral ligaments and reduce bleeding. If bleeding occurs from the lateral ligaments, blood can flow into the retrorectal space, reducing contamination of the lateral operative field. As a result, the RMD procedure leads to less blood loss and shorter operation durations, especially in challenging low rectal cancer surgeries.

Furthermore, with a clear operating field and full exposure of the surgical space, it is possible to preserve the pelvic autonomic nervous system, which includes the superior and inferior hypogastric plexuses. The superior hypogastric plexuses are formed from aortic nerve and merge into the left and right hypogastric nerves. The inferior hypogastric plexuses are mixed sympathetic and parasympathetic ganglionated plexuses. They receive signals from preganglionic axons, visceral afferent axons and postganglionic vasoconstrictor axons from the sacral sympathetic chain, and provide innervation to pelvic organs such as the prostate, seminal vesicle, and erectile structures in the perineum 11. All pelvic nerves travel in the plane between the endopelvic fascia and the peritoneum. Radical rectal cancer surgery carries a risk of damaging these autonomic nerves, which can lead to bowel, urinary bladder, or sexual dysfunction postoperatively. Precise dissection through specific fascia planes can help reduce the risk of nerve damage and dysfunction. Damage to nerve branches from the S3-S4 spinal segments can result in urinary retention 12. The incidence rate of urinary dysfunction after radical rectal cancer resection is about 15-25%. However, with the protection of pelvic autonomic nerve, this can be reduced to 0-12% 13. Similarly, low anterior resection syndrome, characterized by bowel dysfunction, is often reported with standard TME technique involving the removal of perirectal tissue 14. Up to 80% of patients may experience some degree of bowel dysfunction 15. However, bowel functions can be improved with the protection of pelvic autonomic nerve. Our study demonstrated that the IPSS score and the LARS score of the RMD group were lower than those of the TRM group, indicating better urinary and bowel function preservation in the RMD group.

Injures of the pudendal nerve, pelvic plexus, NVB and its branches can significantly impact postoperative erectile function in males. The hypogastric nerve also sends branches to the prostate and seminal glands. Nerve fibers run in the plane anterior to Denonvilliers' fascia, which is a distinct double-layered fascia between the rectum and seminal glands. Heald proposed that the TME technique for rectal cancer should involve dissection anterior to Denonvilliers' fascia 16. Resection of Denonvilliers' fascia can lead to sexual dysfunction in 8-28% of patients 17. In narrow male pelvis surgeries, the operative field is often insufficiently exposed, and the anatomical layers may not be clearly defined, especially after chemoradiotherapy. By performing lateral and posterior rectal mobilization using RMD procedures, the anterior Denovilliers' fascia can be easily exposed. The NVB at the 2 o'clock and 10 o'clock positions, the pelvic plexus, and the hypogastric nerve can be effectively protected in the plane of the seminal vesicle. With the protection of the pelvic plexus branches, the incidence of sexual dysfunction can be reduced to 3%-14%, which is consistent with our study results 13.

The incidence of complications was lower in the RMD group compared to the TRM group, although there was no statistical difference in the composition of complications, such as anastomotic leak and ileus between the two groups. It is important to note that all patients were operated on in the same center by the same group of experienced colorectal surgeons. Both groups were similar in terms of pathological CRM positivity and tumor stage. During the follow-up period, there was also no statistical difference in terms of local recurrence and one-year disease-free survival. However, the RMD group had a higher rate of complete mesorectum and specimen and harvested more peri-intestinal lymph nodes. Longer follow-up time is need to fully understand the impact of RMD on tumor recurrence. Our study patients are currently being followed up, and we expect to obtain long-term outcome data in the future.

Indeed, our study has some limitations, including being a single-center study. However, we have described a practical procedure and demonstrated that it can effectively reduce blood loss, operative durations and hospital stay without increasing postoperative complications. Furthermore, the RMD technique preserves better urogenital and defecation function compared to traditional approaches, which is particularly important given the increasing focus on functional recovery and quality of life in rectal cancer patients. Based on our findings, we suggest that RMD is a safe and feasible option for male patients with middle and low rectal cancer. The results of our clinical trial can provide valuable guidance and support for surgeons in their decision-making process.

## Supplementary Material

Supplementary video 1.

Supplementary video 2.

## Figures and Tables

**Table 1:** Baseline clinical characteristics of patients.

Characteristics	RMD group	TRM group	*P* value
**n**	53	50	
**Age (years)**	58.8 ± 9.6	58.6 ± 6.9	0.90
**BMI (kg/m^2^)**	24.6 ± 2.7	24.0 ± 2.4	0.19
**Previous history**			
** Diabetes**	9 (17.0%)	7 (14.0%)	0.68
** Coronary heart disease**	7 (13.2)	8 (16.0%)	0.69
** Abdominal operation**	5 (9.4%)	3 (6.0%)	0.52
**Tumor characteristic**			
** Tumor size**	3.0 ± 1.0	2.9 ± 0.8	0.53
**T category**			
** T2**	15 (28.3%)	10 (20.0%)	
** T3-4**	38 (71.7)	40 (80.0%)	0.33
**N category**			
** N0**	18 (34.0%)	18 (36.0%)	
** N+**	35 (66.0%)	32 (64.0%)	0.83
** Distance from the anal verge**	6.7 ± 1.7	6.9 ± 1.9	0.62
** Low rectal cancer**	20 (37.7%)	16 (32.0%)	
** Middle rectal cancer**	33 (62.3%)	34 (68.0%)	0.54
** Anterior wall**	12 (22.6%)	9 (18.0%)	
**Lateral and posterior wall**	41 (77.4%)	41 (82.0%)	0.56
**XELOX**	25 (47.2%)	22 (44.0%)	
**Capecitabine**	28 (52.8%)	28 (56.0%)	0.75
**Radiation (45Gy)**	53 (100%)	50 (100%)	

Data are mean ± SD. RMD, rectal modular dissection. TRM, traditional rectal mobilization.

**Table 2:** Perioperative outcomes and pathological characteristics.

Characteristics	RMD group	TRM group	*P value*
**n**	53	50	
**Duration of operation (minutes)**	127.9 ± 27.4	143.8 ± 24.6	0.00
**Blood loss (ml)**	35.9 ± 20.2	83.6 ± 27.5	0.00
**Preventive stoma**	20 (37.7%)	16 (32.0%)	0.54
**Complications**	4 (7.5%)	11 (22.0%)	0.04
**Anastomotic leakage**	2 (3.8%)	4 (8.0%)	
**Stoma-related complications**	0	1 (2.0%)	
**Urine retention**	2 (3.8%)	5 (10.0%)	
**Ileus**	0	1 (2.0%)	0.83
**Clavien-Dindo grade**			
**I-II**	4 (7.5%)	8 (16.0%)	
**III-IV**	0	3 (6.0%)	0.24
**Duration of hospital stay, days**	7.0 ± 1.3	7.6 ± 1.7	0.04

Data are mean ± SD. RMD, rectal modular dissection. TRM, traditional rectal mobilization.

**Table 3:** Pathological properties of two groups.

1. Characteristics	2. RMD group	3. TRM group	*4. P* value
**5. n**	6. 53	7. 50	8.
**9. CRM positive**	10. 1 (1.9%)	11. 5 (10.0%)	12. 0.08
**13. Completeness of mesorectum**	14. 52 (98.1%)	15. 43 (86.0%)	16. 0.02
**17. Specimen grading**	18.	19.	20.
**21. Complete**	22. 51 (96.2%)	23. 41 (82.0%)	24. 0.02
**25. Nearly complete**	26. 1 (1.9%)	27. 3 (6.0%)	28.
**29. incomplete**	30. 1 (1.9%)	31. 6 (12.0%)	32. 0.66
**33. Harvested peri-intestinal lymph nodes**	34. 14.1 ± 5.5	35. 12.0 ± 4.2	36. 0.03
**37. Tumor differentiation**	38.	39.	40.
**41. Well**	42. 11 (20.8%)	43. 14 (28.0%)	44.
**45. Moderate**	46. 32 (60.4%)	47. 30 (60.0%)	48.
**49. Poor**	50. 10 (18.9%)	51. 6 (12.0%)	52. 0.51
**53. Pathological T category**	54.	55.	56.
**57. T1-2**	58. 39 (73.6%)	59. 38 (76.0%)	60.
**61. T3-4**	62. 14 (26.4%)	63. 12 (24.0%)	64. 0.78
**65. Pathological N category**	66.	67.	68.
**69. N0**	70. 46 (86.8%)	71. 42 (84.0%)	72.
**73. N+**	74. 7 (13.2%)	75. 8 (16.0%)	76. 0.69

Data are mean ± SD. RMD, rectal modular dissection. TRM, traditional rectal mobilization.

**Table 4:** The long-term functional outcomes.

Characteristics	RMD group	TRM group	*P*
**n**	53	50	
**Urethral functions (IPSS)**			
** Before surgery**	1.4 ± 1.3	1.0 ± 1.2	0.16
** After surgery**	2.4 ± 1.7	3.5 ± 2.7	0.01
**Erectile functions (IIEF-5)**			
** Before surgery**	19.9 ± 2.4	20.5 ± 1.6	0.16
** After surgery**	19.8 ± 3.2	17.4 ± 1.7	0.00
**Bowel functions (LARS)**			
** Before surgery**	0.4 ± 1.6	0.7 ± 1.8	0.51
** After surgery**	14.5 ± 7.7	21.5 ± 7.0	0.00

Data are mean ± SD. RMD, rectal modular dissection. TRM, traditional rectal mobilization. IPSS, International Prostate Symptom Score. IIEF-5, International Index of Erectile Function scale. low anterior resection syndrome (LARS) score
